# Supplementary material for: VirSorter: mining viral signal from microbial genomic data
Source: PeerJ. 2015 May 28;3:e985. doi: 10.7717/peerj.985 (PMC4451026; doi:10.7717/peerj.985)
Supplement: Figure S1 [file peerj-03-985-s002.pdf]

Running VirSorter in iPlant Discovery Environment

1. iPlant Registration

To access VirSorter on iPlant, you will first need to create a (free) account. This can be done at this address: https://user.iplantcollaborative.org/register/ (help can be found at https://pods.iplantcollaborative.org/wiki/display/start/Registering+for+and+Managing+your+iPlant+Account).

2. Login to the Discovery Environment

The Discovery Environment is the section of iPlant hosting VirSorter, and can be found at https://de.iplantcollaborative.org/de/

3. Upload your data

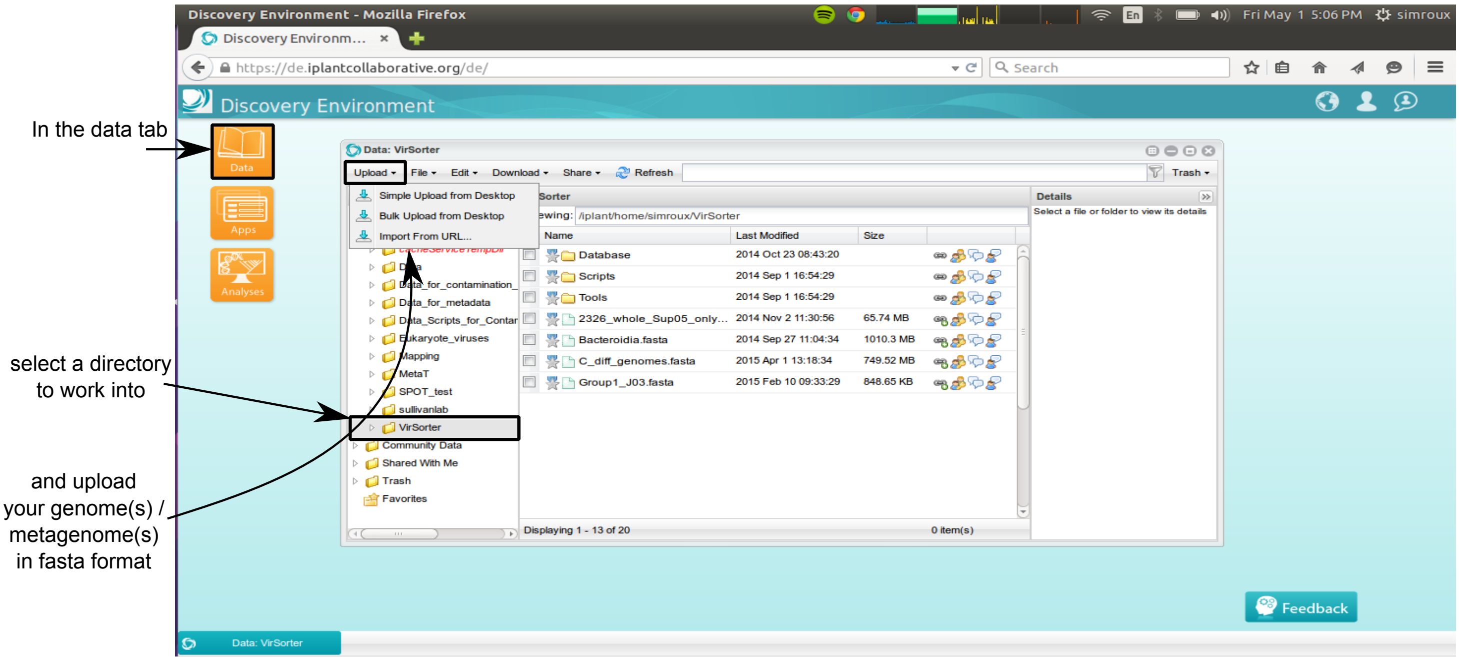

4. Select VirSorter parameters and launch the computation

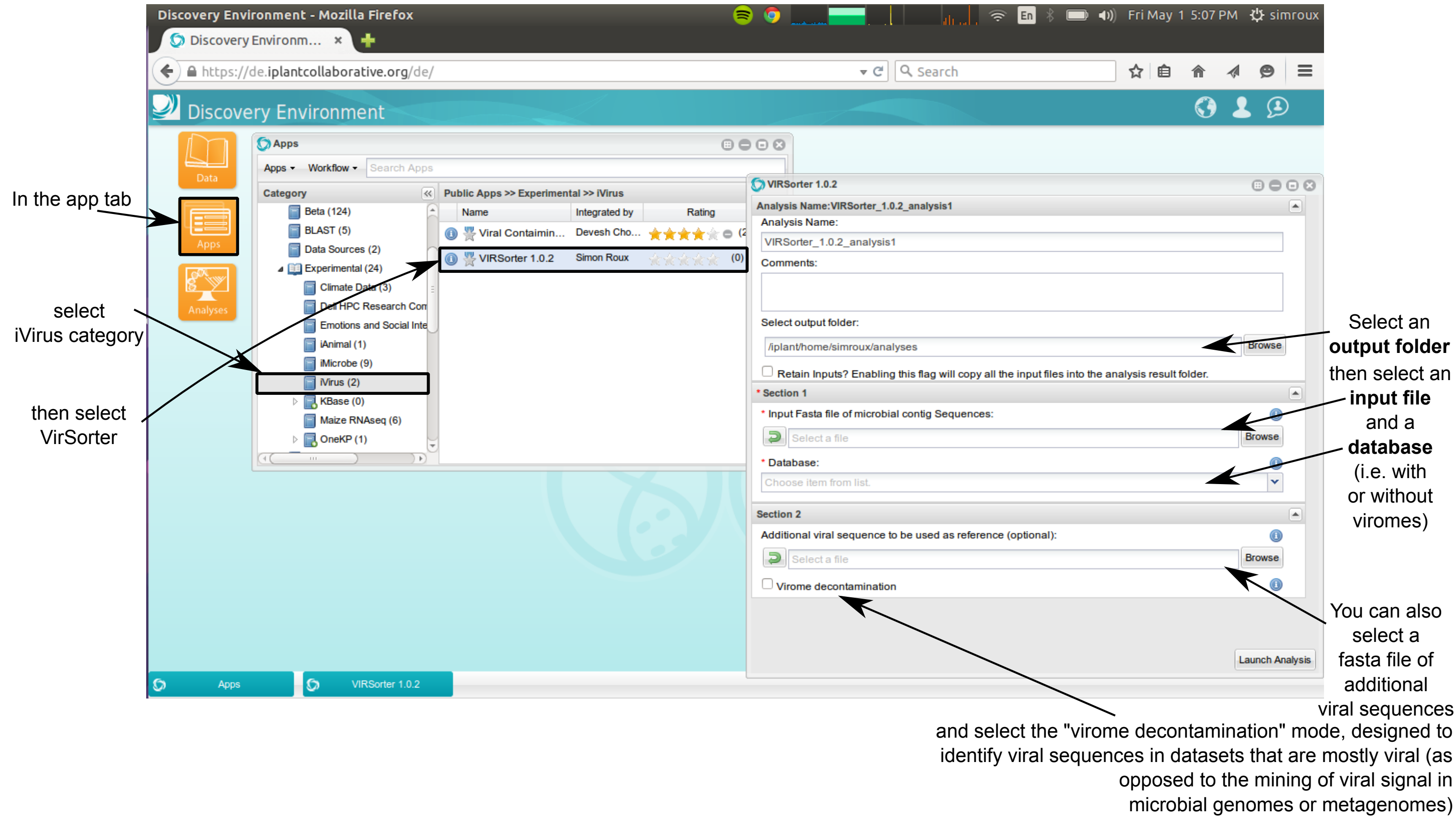

5.Retrieve VirSorter results

Notifications will indicate when the job is (i) submitted, (ii) running, and (iii) completed

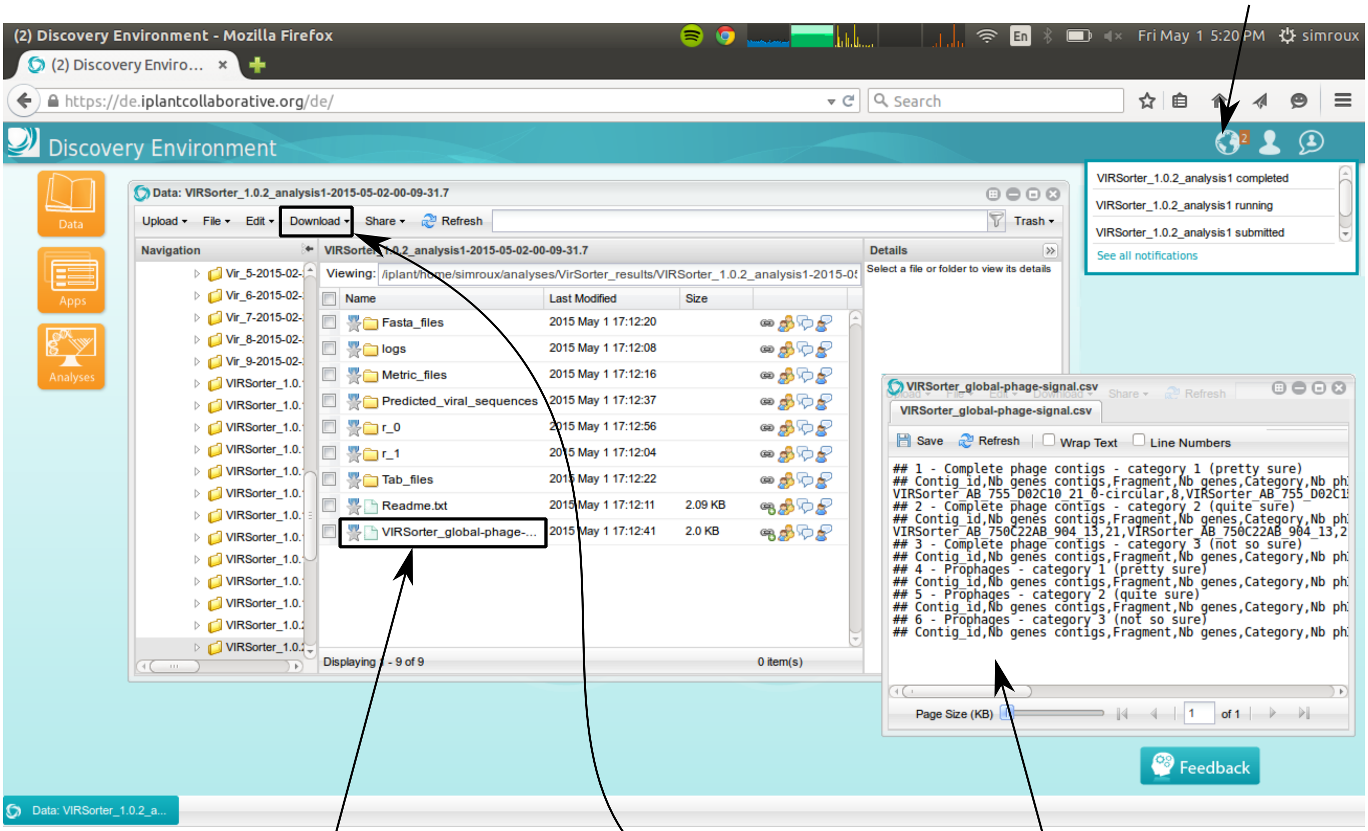

Once completed, the output directory will include the main csv output file, listing all sequences detected as viral with their associated category and metrics, alongside the other result files stored into different sub-directories.

Result files can be opened in the Discovery Environment or downloaded for further processing

See the wiki page of VirSorter for a more complete description of the result files:
https://pods.iplantcollaborative.org/wiki/display/DEapps/VIRSorter+1.0.2
